# The Value of Circulating Tumor HPV DNA in Head and Neck Squamous Cell Cancer: A Review †

**DOI:** 10.3390/diagnostics15212708

**Published:** 2025-10-26

**Authors:** Rüveyda Dok, Sandra Nuyts, Fernando Lopez, Carol Bradford, Arlene A. Forastiere, Primož Strojan, Abbas Agaimy, Göran Stenman, Fernanda V. Mariano, Ilmo Leivo, Karthik N. Rao, Michelle Williams, Avraham Eisbruch, Nabil F. Saba, Alfio Ferlito

**Affiliations:** 1 Laboratory of Experimental Radiotherapy, Department of Oncology, KU Leuven, University of Leuven, 3000 Leuven, Belgium; ruveyda.dok@kuleuven.be; 2 Department of Radiation Oncology, Leuven Cancer Institute, UZ Leuven, 3000 Leuven, Belgium; 3 ENT and Head and Neck Department, Hospital Universitario Central de Asturias, 33011 Oviedo, Spain; fernandolopezphd@gmail.com; 4 Department of Otolaryngology-Head and Neck Surgery, The Ohio State University College of Medicine, Columbus, OH 43210, USA; carol.bradford@osumc.edu; 5 The Sidney Kimmel Comprehensive Cancer Center at Johns Hopkins, Baltimore, MD 21231, USA; af@jhmi.edu; 6 Department of Radiotherapy, Institute of Oncology Ljubljana, Faculty of Medicine, University of Ljubljana, 1000 Ljubljana, Slovenia; pstrojan@onko-i.si; 7 Comprehensive Cancer Center (CCC) Erlangen-EMN, Institute of Pathology, University Hospital, Friedrich-Alexander University Erlangen-Nürnberg (FAU), 91054 Erlangen, Germany; al_ijaimy@yahoo.com; 8 Department of Laboratory Medicine/Pathology, Sahlgrenska Center for Cancer Research, Sahlgrenska University Hospital, University of Gothenburg, 405 30 Gothenburg, Sweden; goran.stenman@llcr.med.gu.se; 9 Department of Pathology, School of Medical Sciences, University of Campinas (UNICAMP), Campinas 13083-970, SP, Brazil; fevimariano@gmail.com; 10 Institute of Biomedicine, Pathology, University of Turku, Turku University Hospital, 20520 Turku, Finland; ilmo.leivo@utu.fi; 11 Department of Head and Neck Oncology, Sri Shankara Cancer Hospital and Research Centre, Bangalore 560 004, India; karthik.nag.rao@gmail.com; 12 Department of Anatomical Pathology, University of Texas MD Anderson Cancer Center, Houston, TX 77030, USA; mdwillia@mdanderson.org; 13 Department of Radiation Oncology, University of Michigan, Ann Arbor, MI 48109, USA; eisbruch@med.umich.edu; 14 Department of Hematology and Medical Oncology, Winship Cancer Institute, Emory University, Atlanta, GA 30322, USA; nfsaba@emory.edu; 15 The International Head and Neck Scientific Group, 35100 Padua, Italy; profalfioferlito@gmail.com

**Keywords:** head and neck squamous cell carcinoma, human papillomavirus, ctDNA, ctHPV-DNA

## Abstract

Human papillomavirus (HPV)-related oropharyngeal squamous cell carcinomas (OPSCC) represent a distinct subgroup of head and neck squamous cell carcinoma (HNSCC) characterized by better prognosis and increased radiosensitivity compared to HPV-negative OPSCC. However, current diagnostic and monitoring methods, including tissue biopsies and imaging, are insufficient for precise risk stratification and early detection of recurrence, leading to challenges in treatment de-escalation and surveillance strategies. Circulating tumor HPV DNA (ctHPV-DNA) has emerged as a promising minimally invasive biomarker that offers tumor-specific detection and monitoring capabilities, potentially transforming the management of HPV-related OPSCC through early disease detection, treatment response assessment, recurrence surveillance stratification, and disease monitoring. Despite encouraging results from early clinical studies, current use is limited to trial settings. Large-scale prospective studies are needed to validate its clinical utility and determine whether early ctHPV-DNA testing can improve patient outcome while reducing treatment related morbidity. This review outlines the biological rationale, technological approaches, and current clinical evidence for ctHPV-DNA in HPV-related OPSCC, emphasizing its potential role in treatment monitoring and surveillance.

## 1. Introduction

Oropharyngeal squamous cell carcinoma (OPSCC) is a distinct subgroup of head and neck squamous cell carcinoma (HNSCC), encompassing cancers of the base of the tongue, soft palate, uvula, and tonsils [1,2]. In addition to the traditional etiological factors such as alcohol and tobacco, human papillomavirus (HPV) infection has emerged as an important risk factor [3,4,5]. Although HPV-related tumors generally have a better prognosis and greater sensitivity to radiation than HPV-negative tumors, the standard treatment approach remains largely the same regardless of HPV status. Most patients still receive radiotherapy (RT) or concurrent chemotherapy (CRT), which can lead to long-term toxicity and diminished quality of life (QoL) [4,6,7,8,9].

De-escalation treatment strategies have been explored to reduce the RT-related long-term toxicities, while preserving treatment efficacy in patients with HPV-related OPSCC [6,7]. However, the outcomes of these trials have been inconsistent, largely due to the heterogeneous treatment responses. Although HPV testing has clinical value in certain settings such as carcinoma of unknown primary or transoral (robotic) surgery for early stage HPV-related OPSCC [8,9]. Current patient selection based on p16 staining and/or HPV DNA testing is insufficient to identify patients for treatment adaptation [6,7,10].

Moreover, standard diagnostic and follow-up tools, including clinical examination and imaging, lack the sensitivity to detect minimal residual disease (MRD). Liquid biopsies (LB) approaches offer a minimally invasive alternative for disease monitoring [11,12,13]. Among these, circulating tumor associated HPV-DNA (ctHPV-DNA) has gained interest in HPV-related OPSCC disease as a promising diagnostic tool due to its biological relevance, tumor specificity, and potential to detect and monitor treatment responses [11,14,15].

In this review, we provide an overview of the use of ctHPV-DNA in the management of HPV-related OPSCC, with focus on its role in treatment monitoring, and disease surveillance.

## 2. Cell Free DNA as a Biomarker in HNSCC

Cell-free DNA (cfDNA) refers to short DNA fragments (<200 bp) originating from nucleosomes that are released into the bloodstream through apoptosis, necrosis, or active secretion. It can also be detected in other body fluids, including saliva, urine, stool, and cerebrospinal fluid. In healthy individuals, cfDNA predominantly originates from leukocytes, but it can also come from other normal cells. In cancer patients, a fraction of the cfDNA originates from tumor cells, and this fraction is called circulating tumor DNA (ctDNA). ctDNA harbors tumor-specific genomic and epigenetic alterations, making it a valuable cancer biomarker [11,12,13,16,17,18].

ctDNA detection and analysis relies on two main strategies: targeted and genome-wide approaches. In targeted approaches, specific genomic regions are sequenced at high depth to detect tumor-specific mutations or alterations. In genome-wide approaches, ctDNA is analyzed in an unbiased manner for fragmentation patterns, structural variants, methylation signatures, and repetitive elements. While genome-wide approaches provide a broader picture of the tumor, they are technically more complex and costly [19,20,21,22,23].

Recent discoveries in ultrashort fragment analysis resulted in a paradigm shift in LB technology, further increasing the detection sensitivity and specificity of these technologies. Studies from 2024 demonstrate that conventional ctDNA assays miss ultrashort fragments (<50 bp), which constitute the majority of transrenal ctDNA in urine samples [24]. This discovery has led to the development of ultrashort droplet digital PCR (dPCR) assays that increase detection rates by 2.6-fold compared to conventional approaches, offering new possibilities for at-home specimen collection and improved patient access to monitoring [24].

## 3. ctHPV-DNA Detection as a Biomarker for HPV-Related OPSCC

Virally mediated cancers such as HPV-associated OPSCC or Epstein–Barr virus (EBV)-related nasopharyngeal carcinomas have the advantage that viral and human genomes are distinctly different, and viral oncogenes are well conserved in tumor tissues. This biological specificity together with advances in molecular diagnostics particularly in dPCR and next-generation sequencing (NGS), has led to the development of multiple assays to detect ctHPV-DNA [6,10,25,26,27,28,29,30,31,32,33,34,35,36,37,38,39,40,41,42]. These improvements together with the biological specificity of HPV, make ctHPV-DNA a promising biomarker for treatment monitoring, early recurrence detection, and risk-adapted management of HPV-related OPSCC.

## 4. Role of ctHPV-DNA in Detection, Treatment Monitoring, and Surveillance

### 4.1. ctHPV-DNA Detection

Unlike cervical cancer, the primary malignancy caused by HPV infection, there are currently no established screening programs for other HPV-related cancers, including HPV-associated OPSCC. The lack of treatable precancerous lesions, combined with the occult nature of early-stage tumors, poses significant challenges to early detection and hinders the development of effective screening programs aimed at primary prevention [3,4,5,40,43]. Targeted screening of high-risk individuals who are likely to benefit would be a more beneficial approach, but no suitable high risk target population has been defined yet for OPSCC [4,5,6,16,18,43]. Early detection of HPV-related OPSCC is further impaired by the fact that early tumors in tonsillar crypts are difficult to inspect.

ctHPV-DNA can detect HPV-related OPSCC with moderate to high sensitivity and high specificity [6,11,13,14,16,17,18,26,27,28,29,30,31,32,33,34,35,36,37,39,41,42,43,44,45,46,47]. The choice of assay technology is critical to test performance in HPV-related OPSCC. Studies using dPCR and NGS have shown superior sensitivity, ranging from 82 to 98% for plasma ctDNA detection compared to quantitative PCR (qPCR) methods. This finding was confirmed by a recent meta-analysis of 21 studies involving 1436 OPSCC patients (Table 1), with pooled sensitivity and specificity estimates of 0.66 (95% CI, 0.58–0.74) and 0.94 (95% CI, 0.59–0.99) for qPCR, 0.89 (95% CI, 0.78–0.94) and 0.97 (95% CI, 0.94–0.99) for dPCR, and 0.91 (95% CI, 0.81–0.96) and 0.97 (95% CI, 0.90–0.99) for NGS, respectively [42].

### 4.2. Treatment Monitoring and Surveillance

ctHPV-DNA detection is particularly relevant in treatment monitoring and surveillance settings in OPSCC patients with pathologically confirmed HPV-positive disease. According to the National Comprehensive Cancer Network (NCCN) guidelines, post-treatment follow-up for HNSCC involves regular scheduled physical examinations and baseline post-treatment imaging within 6 months. In practice, surveillance guidelines vary between institutions but typically include imaging approximately 3 to 6 month-months post-treatment. There is less agreement regarding routine follow-up, especially radiological examinations beyond 2–3 years due to the rarity of recurrence in asymptomatic patients and lack of evidence of the survival benefit from extended imaging [33,48]. In HPV-related OPSCC, most recurrences are often clinically undetectable and are only identified on imaging during follow-up or diagnosed based on patient-reported symptoms [33]. Hence, very few recurrences are being detected by routine physical exams alone. Moreover, intensifying imaging schedules as a surveillance strategy is not a viable solution, since there is no evidence that it improves patient outcomes [14,33,48].

Several studies confirmed a strong positive correlation between imaging findings and the ctDNA levels in recurrent HPV-positive OPSCC with up to 100% negative predictive value (NPV) of HPV ctDNA testing to rule out tumor recurrence [49,50]. Particularly in doubtful cases, e.g., when PET results are inconclusive and difficult to interpret, ctDNA testing appears to be a valuable aid for physicians [51].

ctHPV-DNA detection and particularly the use of tumor tissue-modified viral (TTMV)-HPV DNA (NavDx, Naveris, Waltham, MA, USA) has shown promise in surveillance settings. TTMV-HPV DNA is a CLIA-accredited advanced laboratory-developed test (ALDT) available in the US. It is based on a dPCR technology and targets multiple high-risk HPV subtypes including HPV16/18/31/33/35. It has achieved analytical validation with limits of detection ranging from 0.56 to 1.31 copies/μL for the five HPV types [52].

A meta-analysis of dPCR-based ctHPV-DNA testing, including TTMV-HPVDNA studies, in the post-treatment setting showed a pooled sensitivity of ≈86% and a specificity of ≈96% [53]. Most apparent false positives are detected in young women with unknown cervical HPV status [28,33]. However, caution is warranted with these numbers since the number of controls are limited. TTMV-HPV testing in recurrent settings have reported positive predictive values (PPV) ranging from 93 to 100% [53. It should be noted that Chera et al. reported only a PPV of 53% for a single positive test, which further increased to 94% after two consecutive positive tests [31,33,36]. NPV of ctHPV-DNA testing ranges from 89 to 100% [33. Although ctHPV-DNA assays show high reliability, a retrospective analysis of 399 OPSCC patients by Ferrandino et al. reported that approximately 1 in 10 recurrences may be false negative [29].

ctHPV-DNA dynamics have been correlated with treatment outcome and prognosis and can be predictive of recurrence before biopsy-proven diagnosis, with lead times up to 18 months [14,29,31,33,36,38,39,41,42,53,54].

Chera et al. reported that patients with ctHPV-DNA clearance, which was defined as high baseline ctHPV-DNA (>200 copies/mL) and >95% clearance by week 4 of CRT, had 100% regional disease-free survival. In contrast, patients with an unfavorable ctHPV-DNA clearance profile, combined with adverse clinical risk factors (>10 pack-years smoking history or T4 tumors) showed a recurrent rate of 35% after CRT [28]. The authors link the association of low copy, low tumor burden, and worse prognosis to potentially higher HPV integration [28]. In contrast, Adrian et al. reported a positive correlation between ctHPV-DNA levels and tumor burden (R = 0.39, *p* ≤ 0.001) [55]. This was further confirmed by the study of Cao et al. in which early ctDNA kinetics were found to predict response to definitive CRT in p16-positive stage III oropharyngeal tumors. Low pre-treatment ctDNA and an early increase in ctDNA at week 2 (compared to baseline) correlated with better freedom from progression. Multivariate analysis showed that ctDNA and imaging metrics were comparable in predicting freedom from progression [56].

In neoadjuvant chemotherapy setting, rapid early ctHPV-DNA clearance after one cycle of neoadjuvant therapy predicted radiographic deep response (≥50% tumor shrinkage per RECIST v1.1), whereas detection of ctHPV-DNA 3 months or later after treatment was associated with worse progression-free and overall survival in 46 patients with non-metastatic HPV-positive OPSCC. In this study, sensitivity, specificity, and PPV and NPV of longitudinal ctHPV-DNA were 100% [57].

A limitation of ctHPV-DNA-based assays was noted in a recent analysis of arm A of the NCT05307939 trial [54], in which the investigators used TTMV-HPV DNA to select patients for active surveillance, omitting or delaying adjuvant RT until patients developed detectable HPV ctDNA. Patients were eligible for the study if they had a preoperative TTMV-HPV DNA score of ≥50 fragments/mL, underwent complete surgical resection of all gross disease confirmed by post-operative MRI, had at least one pathological risk factor for adjuvant therapy, and had a negative post-operative TTMV-HPV DNA at 2–6 weeks post-surgery. The active surveillance consisted of TTMV-HPV DNA testing, imaging, and physical exams every 3 months. Patients received adjuvant RT if they developed detectable TTMV-HPV DNA without evidence of gross disease. The primary endpoint was defined as ≥85% of patients remaining free of gross disease progression at 1 year. With 12 patients enrolled, the trial would fail to meet its endpoint if three or more events occurred. During surveillance, TTMV-HPV DNA was detected in 1 of 12 patients, 6 months post-surgery without evidence of gross disease. Additionally, three patients developed radiographically detected progression at 6 months, in two cases recurrence occurred synchronously with ctHPV-DNA detection, and in one case, before ctHPV-DNA detection. Ultimately, the study did not meet its primary endpoint and was unable to reliably predict radiographic recurrence. The small sample size may have contributed to this outcome. More importantly, reliance on ctHPV-DNA without integration of clinical parameters appears insufficient. The authors also concluded that additional clinical factors are required for the selection of patients for active surveillance and initiation of delayed adjuvant therapy prior to recurrence [54].

On the contrary, a secondary analysis of the DART (De-escalated Adjuvant Radiation Therapy) phase 3 randomized clinical trial confirmed that patients with HPV-positive OPSCC who had detectable ctHPV-DNA 3 months after surgery had a higher risk of disease progression [58]. This finding also suggests the potential of the ctHPV-DNA testing for selecting patients for treatment de-escalation and identifying patients with disease progression early in surveillance.

Although not exclusively in HPV-positive OPSCC, Zhang et al. confirmed the utility of ctDNA as a decision-guided biomarker in patients with locally advanced oral cavity, oro-/hypopharyngeal or laryngeal cancer [59]. Following neoadjuvant chemoimmunotherapy and surgery that resulted in a major pathological response, 69 patients received tumor-informed ctDNA-guided postoperative treatment and 118 received standard pathology-guided adjuvant therapy. Compared to traditional protocol, significantly fewer patients in the ctDNA-guided group received postoperative chemoradiation (27.5% vs. 42.4%, *p* = 0.042). ctDNA-guided management showed a 15% reduction in locoregional recurrence risk (adjusted hazard ratio of 0.85 with 95%CI between 0.70 and 0.94, *p* = 0.013) and adjuvant CRT benefit was more pronounced in ctDNA-positive patients [59].

## 5. Future Perspectives

The incidence of OPSCC is rising in Europe, the UK, and the US, largely driven by the increase in HPV-related disease. Data from both UK and the US show that the incidence of OPSCC in men has surpassed that of cervical cancer in women. Globally, approximately 33% of OPSCC is related to HPV. However, the prevalence varies considerably depending on the geographical region, ranging from 25% to 80% [3,4,43,60,61].

The implementation of vaccination programs is expected to reduce the incidence of OPSCC in the long-term. However, this benefit may only become evident after several decades [3,4,60,61]. Until then improving treatment outcome and surveillance remain important. Treatment adaptation or surveillance strategies will only be beneficial in appropriately selected patients. In the meantime, surveillance and management of HNSCC and OPSCC remain suboptimal, leading to discussions regarding the clinical value and use of early treatment response detection strategies.

**Figure 1:**
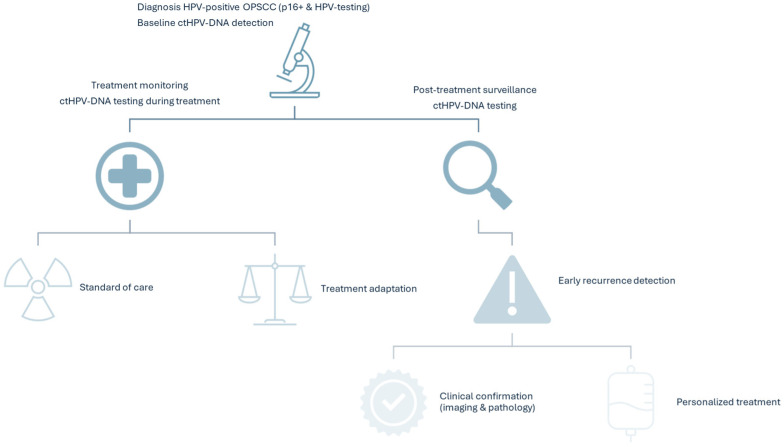
Potential use of ctHPV-DNA testing in the management of HNSCC cancers.

ctHPV-DNA testing has especially a potential in early recurrence detection and treatment stratification by identification and selection of low-risk patients for safe de-escalation (Figure 1) [3,4,11,14,15,33,38,43,60,62]. Importantly, its use as a molecular surveillance tool appears to inspire a high level of confidence significantly reduces the distress levels of patients with HPV-positive OPSCC [63].

However, overinterpretation of test results may lead to patient anxiety, unnecessary diagnostic procedures, or overtreatment in cases where transient positivity does not correspond to clinically significant disease. Furthermore, the financial burden of repeated testing and the integration of advanced molecular assays into routine practice may limit accessibility and raise cost-effectiveness concerns.

Moreover, it is important to acknowledge the gap between early detection of molecular markers and their potential to improve clinical outcome. A recent randomized prospective trial for nasopharyngeal carcinoma patients failed to show benefit of adjuvant chemotherapy in patients with detectable post-RT plasma EBV [64], meanwhile early use of pembrolizumab in high-risk HNSCC prolonged progression-free but not overall survival [65].

These findings highlight the need to validate ctHPV-DNA assays in large-scale, prospective, multi-center studies to define clear thresholds for clinical decision-making and develop guidelines to minimize the risks of false positives and overdiagnosis. In addition, it is essential to determine whether early ctHPV-DNA detection can impact patient outcomes. Therefore at present, ctHPV-DNA should be viewed as a complement to imaging or clinical examination and its use should primarily be in clinical trial settings [11,14,15,33,38].

Currently, most of the available data is derived from retrospective observational studies. Several clinical trials evaluating the role of ctHPV-DNA, alone or in combination with cfDNA, for diagnosis, treatment response, and surveillance in HNSCC are ongoing (Table 2). To our knowledge, SURVEILLE-HPV (NCT05582122) remains the only randomized trial currently recruiting to assess ctHPV-DNA as a biomarker for early recurrence in OPSCC.

Although, dPCR appears to be a technology that is relatively easy to implement in clinical practice due to its high specificity and sensitivity comparable to NGS based approaches, with less cost and bioinformatic requirements [11,12,14,38,41,66]. The broader cfDNA field has shown rapid progress driven by improvements in sequencing technologies, the development of advanced bioinformatic pipelines, and the integration of machine learning methods. Tumor-informed assays, which use genomic information from the patient’s own tumor to guide ctDNA detection, have shown improved sensitivity in other cancers and have also demonstrated predictive and prognostic potential in head and neck cancer (Table 3) [11,12,14,59,67]. Adaptation of these assays to HPV-related OPSCC in few studies have shown increased sensitivity [45,47,68,69,70].

Emerging fragmentomic approaches combined with artificial intelligence algorithms are achieving unprecedented sensitivity in early-stage disease detection, with some studies reporting >95% accuracy in identifying MRD when combining mutational analysis with fragment pattern recognition [71]. These artificial intelligence-powered approaches may address the current limitation of false-negative rates in low-tumor-burden patients, a critical gap identified in recent prospective trials [72]. Continued refinement of cfDNA-based approaches could expand early disease or MRD detection and risk and treatment stratification leading to biology-driven treatment adaptation and surveillance across a broader HNSCC patient population.

## 6. Economic Considerations and Implementation Barriers

From implementation perspective, cost-effectiveness analyses reveal that ctHPV-DNA surveillance may reduce healthcare costs when properly implemented. Economic modeling demonstrates potential savings of USD 4239 per patient compared to imaging-based surveillance (USD 8541 vs. USD 12,780), primarily through reduced imaging frequency, earlier intervention and when the assay is performed ≤6 time points per patient [33,66,73].

However, implementation faces significant barriers including inconsistent payer coverage, with many insurers considering LB experimental despite growing clinical evidence [74].

As mentioned before, TTMV-HPV DNA assay (NavDx) has demonstrated high specificity in both diagnostic and surveillance settings in US [52]. However, sensitivity varies, highlighting the need for complementary use alongside existing surveillance methods [52]. Moreover, biological and technical limitations can also impact the assay performance. The ctDNA fraction within the total cfDNA is influenced by tumor burden, anatomical site and tumor biology (e.g., proliferative activity, cell death, and invasiveness). Early-stage or slow-growing tumors shed minimal DNA into the circulation, leading to <0.01% of the total cfDNA, which together with a limited number of genomic copies can reduce the sensitivity and detectability. A recent retrospective analysis in a Southeast Asian population shows that early-stage ctDNA detection rates varied from 66.7 to 93.3% for different solid tumors [75], while detection rate for patients with gliomas is below 10% [76]. In addition, ctDNA alterations are not always tumor-specific, matched normal DNA and tumor-informed assays can help mitigate this limitation [11,12,13,14,16,17,18,33,70,76]. Variable viral load, non-standardized thresholds or testing intervals can further affect assay performance. Pre-analytical factors such as sample handling, extraction methods, and storage conditions can affect ctDNA integrity and test reproducibility [6,11,12,13,14]. Ongoing efforts, including ISO/CEN guidelines for LB implementation, are addressing these technical challenges [77].

Hence, widespread implementation will require specialized laboratory infrastructure, trained personnel, and standardized sample handling protocols. Risk-based reimbursement models may facilitate broader adoption while reducing financial uncertainty for healthcare systems [74].

## 7. Conclusions

Circulating tumor HPV DNA has the potential to shape the management of HPV-related OPSCC, offering potential improvements in early detection, treatment monitoring, and surveillance. The biological specificity of ctDNA and advancements in detection technologies support its clinical utility.

Nevertheless, further large-scale prospective studies are essential to understand the limitations, to define clinical benefits, standardize testing protocols and to determine appropriate timing and thresholds. These studies are essential to confirm whether early ctHPV-DNA testing can improve patient outcome, refine risk stratification and can safely guide treatment adaptation, ultimately leading to better patient care.

## Figures and Tables

**Table 1:** Diagnostic performance of ctHPV-DNA detection methods in OPSCC [42].

Method	Sensitivity (95% CI)	Specificity (95% CI)
qPCR	0.66 (0.58–0.74)	0.94 (0.59–0.99)
dPCR	0.89 (0.78–0.94)	0.97 (0.94–0.99)
NGS	0.91 (0.81–0.96)	0.97 (0.90–0.99)

**Table 2:** Ongoing clinical trials for ctHPV-DNA detection in HNSCC.

NCT Number	Study Title	Recruitment Status	Condition	Sponsor	Study Type
NCT05814549	A Study Using Human Papillomavirus (HPV) DNA Testing to Detect HPV-Related Oropharyngeal Cancer (OPC) (NavDx^®^)	Active, not recruiting	Oropharyngeal Human Papillomavirus-Positive Squamous Cell Carcinoma	Memorial Sloan Kettering Cancer Center/New York, NY, USA	Observational
NCT05539638	The Role of Circulating Tumor DNA in Head and Neck Cancer	Recruiting	Head and Neck Cancer	University of Edinburgh/Edinburgh, UK	Observational
NCT05904327	Circulating Biomarkers in Oropharyngeal Cancers	Active, not recruiting	Oropharyngeal Human Papillomavirus-Positive Squamous Cell Carcinoma	Region Örebro County/Sweden	Observational
NL-OMON56995	Personalized follow-up in HPV related oropharyngeal cancer patients using liquid biopsies	Recruiting	Oropharyngeal Human Papillomavirus-Positive Squamous Cell Carcinoma	Universitair Medisch Centrum Utrecht/Utrecht, The Netherlands	Observational
NCT05307939	A Study on Using Cell-Free Tumor DNA (ctDNA) Testing to Decide When to Start Routine Treatment in People With Human Papilloma Virus (HPV)- Associated Oropharynx Cancer (OPC)	Recruiting	Oropharyngeal Human Papillomavirus-Positive Squamous Cell Carcinoma	Memorial Sloan Kettering Cancer Center/New York, NY, USA	Interventional
NCT05268614	Risk Adapted De-Intensification of Radio-Chemotherapy for Oropharyngeal Squamous Cell Carcinoma (Phase 2)	Recruiting	Oropharyngeal Squamous Cell carcinoma	University of Florida/Gainesville, FL, USA	Interventional
NCT05541016	De-Escalated Adjuvant and Definitive Radiation Therapy Informed by DART 2.0 ctHPV-DNA	Recruiting	Oropharyngeal Human Papillomavirus-Positive Squamous Cell Carcinoma	Mayo Clinic/Rochester, MN, USA	Interventional
NCT06821243	Patients with Human Papillomavirus-associated Head and Neck Cancer for the Discovery of Predictive Biomarkers to Guide Clinical Intervention	Recruiting	Oropharyngeal Human Papillomavirus-Positive Squamous Cell Carcinoma	Regina Elena Cancer Institute/ Rome, Italy	Observational
NCT05582122	SURVEILLE-HPV: Evaluation of HPV16 Circulating DNA as Biomarker to Detect the Recurrence, in Order to Improve Post Therapeutic Surveillance of HPV16-driven Oropharyngeal Cancers	Recruiting	Oropharyngeal Squamous Cell Carcinoma	UNICANCER/Paris, France	Interventional: Randomized
NCT04900623	Risk-adapted Therapy in HPV+ Oropharyngeal Cancer Using Circulating Tumor (ct)HPV DNA Profile—The ReACT Study	Recruiting	Oropharyngeal Squamous Cell Carcinoma	Dana-Farber Cancer Institute/Boston, MA, USA	Interventional
NCT04965792	Post-treatment Surveillance in HPV+ Oropharyngeal SCC	Active, not recruiting	Oropharyngeal Human Papillomavirus-Positive Squamous Cell Carcinoma	Dana-Farber Cancer Institute/Boston, MA, USA	Observational

**Table 3:** Ongoing clinical trials for cfDNA detection in HNSCC.

NCT Number	Study Title	Recruitment Status	Condition	Sponsor/Location	Study Type
NCT06036563	Prospective Screening and Differentiating Common Cancers Using Peripheral Blood Cell-Free DNA Sequencing	Recruiting	Pancancer	Air Force Military Medical University/Xi’an, China	Observational
NCT05366881	cfDNA Assay Prospective Observational Validation for Early Cancer Detection and Minimal Residual Disease (CAMPERR)	Recruiting	Pancancer	Adela Inc./Foster City, CA, USA	Observational
NCT07035587	Diagnosis of Multiple Cancer and Monitoring of Minimal Residual Tumors After Treatment Using Blood and High-Sensitivity Genetic Analysis Techniques	Recruiting	Pancancer	Yonsei University/ Seoul, Republic of Korea	Observational
NCT05685524	Clinical Study of Pan-cancer DNA Methylation Test in Plasma	Unknown	Pancancer	Wuhan Ammunition Life-tech Co. Ltd./ Wuhan, China	Observational
NCT03926468	Liquid Biopsy in Head and Neck Cancer	Unknown	Head and Neck Cancer	Turku University Hospital/ Turku, Finland	Observational
NCT03942380	Cell-free Tumor DNA in Head and Neck Cancer Patients	Unknown	Head and Neck Cancer	Rigshospitalet/ København, Denmark	Interventional
NCT06356272	Oropharynx (OPX) Biomarker Trial	Recruiting	Oropharyngeal Squamous Cell carcinoma	Mayo Clinic/Rochester, MN, USA	Observational
NCT04599309	Real-time Detection of ctDNA and/or HPV DNA in High-risk Locally advanced Head and Neck Squamous Cell Carcinoma	Active, not recruiting	Locally Advanced Head and Neck Carcinoma	University Health Network Toronto/ Toronto, ON, Canada	Observational
NCT05710679	Prediction of Residual Disease by Circulating DNA Detection After Potentiated Radiotherapy for Locally Advanced Head and Neck Cancer (NeckTAR)	Recruiting	Locally Advanced Head and Neck Carcinoma	Centre Jean Perrin/ Clermont-Ferrand, France	Interventional
NCT02245100	Circulating Tumor DNA in Predicting Outcomes in Patients with Stage IV Head and Neck Cancer or Stage III-IV Non-small Cell Lung Cancer	Completed	Head and Neck Cancer and Non-small Cell Lung Cancer	Sidney Kimmel Cancer Center at Thomas Jefferson University/Philadelphia, PA, USA	Observational
NCT04606940	Study of Circulating Tumor DNA (ctDNA) Kinetics in Immuno-oncology (IO-KIN)	Completed	Recurrent of metastatic or advanced Head and Neck Cancer	University Health Network Toronto/ Toronto, ON, Canada	Observational

## Data Availability

No new data were created or analyzed in this study. Data sharing is not applicable to this article.
